# Interaction-induced hopping phase in driven-dissipative coupled photonic microcavities

**DOI:** 10.1038/ncomms11887

**Published:** 2016-06-16

**Authors:** S. R. K. Rodriguez, A. Amo, I. Sagnes, L. Le Gratiet, E. Galopin, A. Lemaître, J. Bloch

**Affiliations:** 1 Laboratoire de Photonique et de Nanostructures (LPN), CNRS, Université Paris-Saclay, route de Nozay, Marcoussis F-91460, France; 2 Physics Department, Ecole Polytechnique, Palaiseau Cedex F-91128, France

## Abstract

The Bose-Hubbard model (BHM) describes bosons hopping across sites and interacting on-site. Inspired by the success of BHM simulators with atoms in optical lattices, proposals for implementing the BHM with photons in coupled nonlinear cavities have recently emerged. Two coupled semiconductor microcavities constitute a model system where the hopping, interaction and decay of exciton polaritons—mixed light-matter quasiparticles—can be engineered in combination with site-selective coherent driving to implement the driven-dissipative two-site optical BHM. Here we explore the interplay of interference and nonlinearity in this system, in a regime where three distinct density profiles can be observed under identical driving conditions. We demonstrate how the phase acquired by polaritons hopping between cavities can be controlled through polariton-polariton interactions. Our results open new perspectives for synthesizing density-dependent gauge fields using polaritons in two-dimensional multicavity systems.

## Abstract (web summary)

Coupled semiconductor microcavities constitute a model system where the hopping, interaction, and decay of exciton polaritons can be engineered. Here, Rodriguez *et al*. show how the phase acquired by polaritons hopping between cavities can be controlled through polariton-polariton interactions.

## Graphical abstract

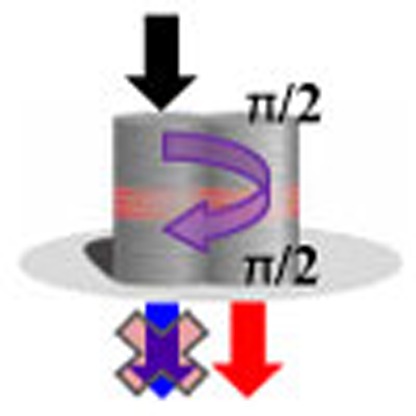

Understanding the emergence of collective phenomena in condensed matter systems is an example of a problem that quantum simulators may address. Ultracold atoms in optical lattices have enabled great progress in this direction^1^. Recently, photonic systems have been proposed for simulating the hopping and interaction of bosonic particles as described by the Bose-Hubbard model (BHM), but in non-equilibrium conditions^2^,^3^,^4^,^5^. In particular, driven-dissipative lattices of coupled nonlinear cavities can display strongly correlated steady-state phases characterized by the number of available stable modes^6^. Moreover, for strong nonlinearities polaritons can crystallize^7^. For the minimal Bose-Hubbard system comprising two sites, that is, a dimer, intriguing quantum interference effects and single photon emission have been predicted^8^,^9^. As we will show, the driven-dissipative Bose-Hubbard dimer (BHD) displays striking phenomena even at the mean-field level due to the interplay of interference, nonlinearity and site-selective coherent driving.

Under time-harmonic driving of one site, the mean fields 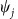 of the driven-dissipative BHD are described by the coupled equations:


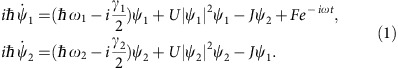


where 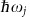 and 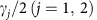 are the on-site energy and decay rate, *J* is the hopping energy and *U* is the interaction energy. *F* and 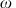 are the driving amplitude and frequency on site *j=*1. The BHD dynamics without driving (*F*=0) has been thoroughly studied with atoms, especially in relation to the self-trapping occurring when the total interaction energy, *U*(*N*_1_+*N*_2_) with 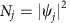 being the mode populations, exceeds *J* (refs 10, 11). For dissipative (for example, photonic) systems, the non-Hermiticity of the BHD Hamiltonian^12^ gives rise to distinct nonlinear phenomena. A dissipation-limited self-trapping time^13^, a dissipation-induced classical to quantum transition^14^ and spontaneous symmetry breaking^15^, have been observed with photons. With coherent driving on one site (*F*≠0), parametric instabilities^16^ and nonclassical correlations^8^,^9^ have been predicted as hopping, interactions and decay compete in setting a stationary state. Despite impressive theoretical efforts in this direction, the driven-dissipative BHD has remained experimentally unreported with photons so far.

An excellent system for implementing the driven-dissipative optical BHD comprises exciton polaritons in coupled semiconductor microcavities. Polaritons are hybrid light-matter quasiparticles formed by strong coupling between cavity photons and quantum well excitons^17^. Polaritons can be confined and coupled by micro-patterning planar cavities, thereby acting on the photonic part of their wavefunction^18^. In this way, Hamiltonians describing molecular orbitals^19^ or particles in lattices^20^ can be implemented. In addition, Kerr nonlinearities associated with the excitonic part of polaritons^21^ yield effective polariton-polariton interactions. Steady-state nonlinearities such as bistability^22^ and polarization multistability^23^ have been observed in single cavities. Accessing the physics of the driven-dissipative non-equilibrium BHM requires spatial coupling of nonlinear cavities, which, in contrast to the coupling of the two polariton spin components^23^, can include many degrees of freedom.

Here we demonstrate spatial multistability in a polariton BHD, and we discover an interaction-induced phase for polaritons hopping between cavities. This nonlinear phase control could enable the realization of non-Hermitian Hamiltonians with density-dependent gauge fields if extended to two-dimensional cavity arrays.

## Results

### Linear regime

**Figure 1:**
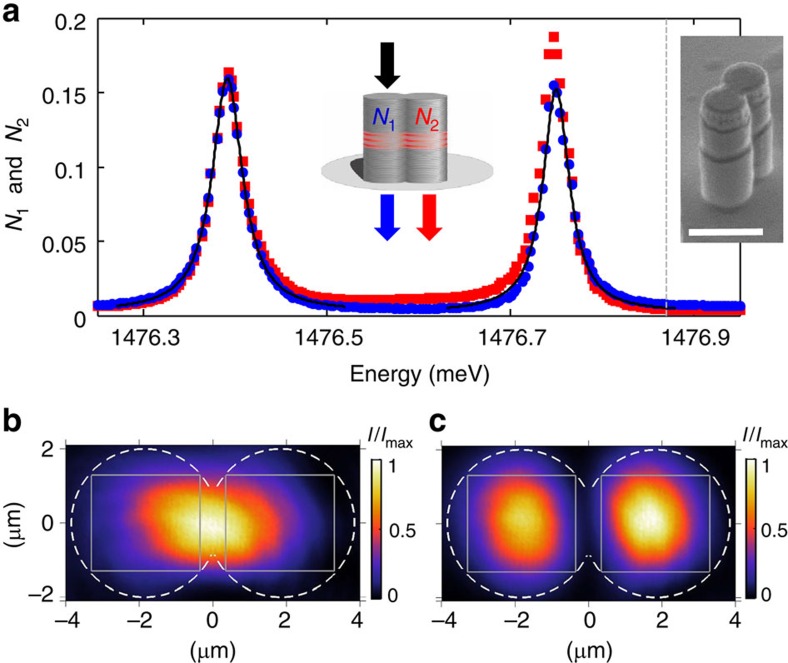
Linear spectrum and eigenmodes. (**a**) Mean polariton number per polariton lifetime in two coupled microcavities under continuous driving of one cavity. The central inset illustrates the spatial and colour code used throughout the manuscript: blue data points for the driven cavity on the left (*N*_1_), and red data points for the undriven cavity on the right (*N*_2_). Black curves are Lorentzian fits. The dashed grey line indicates the driving energy used in Fig. 3 and Fig. 4. The right inset shows a scanning electron micrograph of the structure, where the scale bar denotes 5 μm. (**b**,**c**) show the spatial distribution of the normalized transmitted intensity (*I*/*I*_max_) at the low- and high-energy peaks corresponding to the bonding and antibonding resonances, respectively. The dashed lines delimit the two coupled microcavities. The solid lines indicate the integration area used to evaluate the population in each cavity in this paper.

The two slightly overlapping cavities we investigate are shown in Fig. 1a inset. The coupled cavities behave as a photonic molecule (PM), where strong coupling between polaritons in each cavity forms hybridized states^18^. Figure 1a shows the linear spectrum of the PM. We drive the left cavity with a laser of variable frequency and quantify the cavity populations from spatially resolved transmission measurements (see Methods). The low- and high-energy peaks are the bonding and antibonding resonances of the PM, respectively. At the energy of the antibonding resonance, the exciton fraction of the polaritons is ∼16% (see Supplementary Note 1 and Supplementary Fig. 1 for details). From Lorentzian fits to the spectra (black lines) we extract a bonding-antibonding splitting of 2*J=*358±1 *μ*eV, well above the sum of the linewidths *γ*_*B*_+*γ*_*AB*_=75±3 *μ*eV. Figure 1b shows the bonding mode, with nonzero density at the centre of the dimer reflecting the even parity of the wave function. In contrast, the antibonding mode in Fig. 1c shows suppressed density at the centre due to the odd parity of the wave function.

### Number of modes in the nonlinear regime

**Figure 2:**
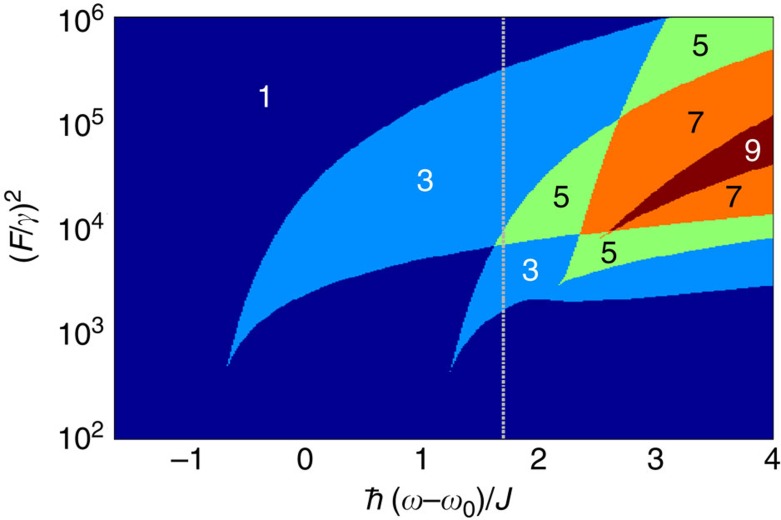
Calculated total number of modes. The total number of modes (stable and unstable) admitted by equation (1) is shown in colour as a function of the energy detuning 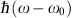 divided by the hopping energy *J*, and of the driving power (*F*/*γ*)^2^, with *F* being the driving amplitude and *γ* the loss rate of each cavity. Identical cavities with eigenfrequency 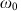 are assumed. The dashed line indicates the driving energy used in Figs 3 and 4.

To illustrate the wealth of nonlinear phenomena expected in the PM according to equation (1), we present in Fig. 2 the calculated total number of modes as a function of the dimensionless frequency detuning 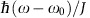 and driving power (*F*/*γ*)^2^. We consider two identical cavities (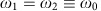 and *γ*_1_=*γ*_2_=*γ*) with repulsive interactions (*U*>0) within each cavity. Figure 2 shows that for weak driving (negligible interactions here achieved for 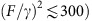 and any 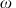, or any power and 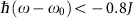, the PM is monostable: there is a single input-output relation. When 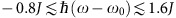, the PM can support up to three modes, two of which are stable, that is, bistability^22^. The monostable and bistable regimes are well known; their observation in our system is presented in Supplementary Notes 2 and 3 and Supplementary Figs 2 and 3. The third and most interesting driving condition is when 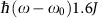, where up to nine modes exist but no more than five are stable. In the following, we show experiments and calculations for 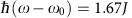 (dashed line in Fig. 2), where we observe three distinct stable modes and one branch displays an interaction-induced hopping phase.

### Tristability and interaction-induced hopping phase

**Figure 3:**
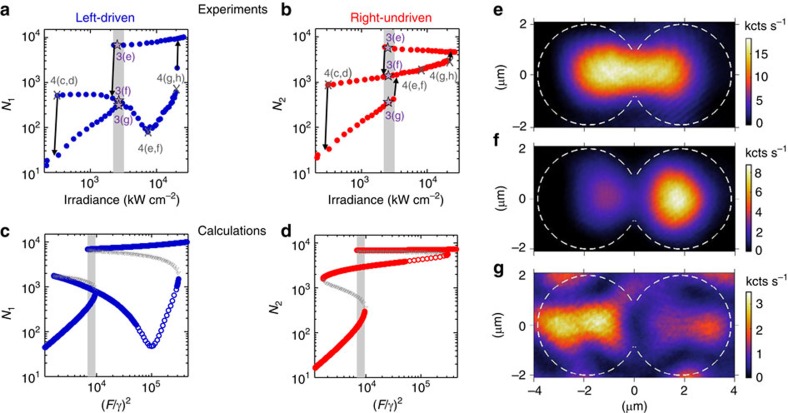
Tristability. (**a**,**b**) show the measured populations in the driven and undriven cavities, respectively, for 1476.87 meV driving energy (dashed line in Fig. 1a). Shaded areas indicate the irradiance range for tristability; (**e**–**g**) show three mode profiles obtained for the same irradiance, as indicated with the purple stars in (**a**,**b**). The grey crosses denote the irradiance corresponding to Fig. 4; (**c**,**d**) show the populations calculated with the model described in the text. Solid curves indicate stable modes, open circles indicate parametrically unstable modes and grey lines indicate unstable modes.

Figure 3a,b shows experiments where the left cavity is driven at an energy of 1476.87 meV (dashed line in Fig. 1a). We observe a pronounced hysteresis in the populations as a function of the irradiance. The hysteresis involves three branches. Changes in density are reversible along each branch. In the shaded region in Fig. 3a,b, the PM is tristable: three different stable density profiles can be observed at the same irradiance. Figure 3e–g illustrates three density profiles at the same irradiance, indicated by the stars in Fig. 3a,b. Which one of these profiles is observed depends on the history of the system, or in which direction the irradiance is scanned.

The nonlinear jumps and the branches in Fig. 3a,b can be understood by comparing the total interaction energy *U*(*N*_1_+*N*_2_) with the energy detuning between the laser and the linear eigenmodes of the system. At the first upwards threshold, the antibonding mode blueshift brings it in resonance with the laser. The signature of the antibonding mode—a suppressed population at the centre of the dimer—can be recognized throughout the middle branches, as illustrated in Fig. 3f. For greater irradiance the second upwards threshold brings the bonding mode in resonance with the laser. This sets the populations into the highest branches, where the features of the bonding mode can be recognized (see the mode profile in Fig. 3e resembling the linear bonding mode in Fig. 1b). We stress that these are all qualitative similarities, since bonding and antibonding are linear eigenmodes of the system. The evolution of the spectrum of the PM in the nonlinear regime is shown in Supplementary Fig. 4 and discussed in Supplementary Note 4.

The measurements in Fig. 3a,b are qualitatively reproduced using equation (1). Figure 3c,d shows calculated populations using parameters deduced from the fits to the linear spectrum (see Methods). Besides the stable modes (solid curves), the calculations show two branches of unstable modes (grey lines) and a regime of parametric instability (open circles) along the middle branch. The unstable branches emerge when a fixed point loses its stability and a new fixed point is created. The parametric instability arises between the first and the second upwards threshold. For a limited power range therein, parametric processes involving the driving laser and the hopping energy *J* (determining the bonding-antibonding splitting) generate new frequencies, that is, a signal and an idler^16^. However, since the new frequencies are generated with a low efficiency, the dominant contribution to the measured populations has the frequency of the driving laser. Consequently, we can continuously and reversibly monitor the cavity populations along the middle branches by varying the irradiance, as would be the case if the entire middle branches were stable. Beyond the qualitative agreement between experiments and calculations along the three observed branches, some quantitative differences are observed. These are likely due to power fluctuations in the driving laser, which make it difficult to access the end points of the branches where instabilities take place. Further differences stem from the fact that in theory, (i) the populations in the driven and undriven cavities are perfectly separable, and (ii) the driving force acts on one cavity only. Both (i) and (ii) are not strictly true in experiments due to the spatial overlap of the cavities and the finite beam waist.

**Figure 4:**
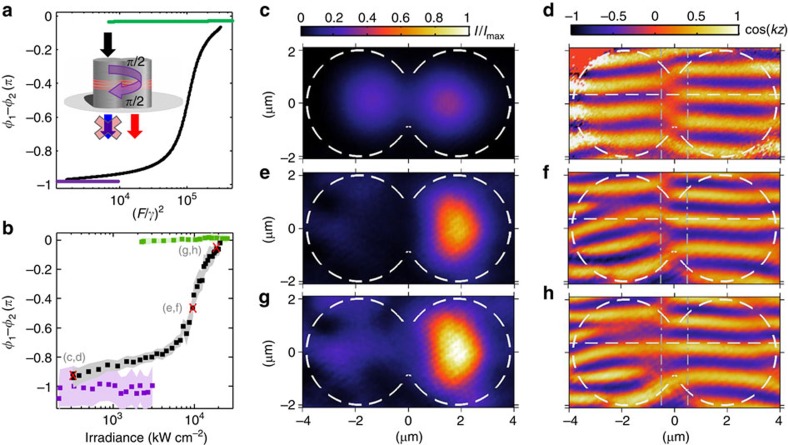
Interaction-induced hopping phase. (**a**) Calculated and (**b**) measured phase difference between the intracavity fields for the three branches experimentally observed in Fig. 3. The phase difference *φ*_1_–*φ*_2_ is shown in purple for the low-density branches, in green for the high-density branches and in black for the middle branches (where driven cavity exhibits a dip in the population). The shaded areas in (**b**) correspond to 2*σ* (≈95%) confidence intervals, that is, two standard deviations, on the fits performed to retrieve the phase of each cavity (see Methods). (**c**–**h**) show three representative density (central panels) and normalized interferogram (right panels) plots along the middle branch. The effective hopping phase *φ*_1_–*φ*_2_ and irradiance corresponding to each one of these three cases is marked by a red cross in (**b**). The colour bar on top of panel (**c**) applies to all three density plots (**c**,**e**,**g**), while the colour bar on top of panel (**d**) applies to all three normalized interferograms in (**d**,**f**,**h**). The vertical dash-dotted lines in (**d**,**f**,**h**) indicate where cosine functions were fitted to the normalized interferogram to retrieve the phase of each cavity. The horizonal dashed lines in (**d**,**f**,**h**) are all at the same position and serve as guides to the eye.

A striking feature in Fig. 3a,c is the pronounced population dip along the middle branch. The occurrence of this dip between the two upwards thresholds and its absence in the undriven cavity suggests that this is an interference effect. To elucidate the underlying mechanism, we calculate in Fig. 4a the difference *φ*_1_–*φ*_2_ between the phases of the field in each cavity (see Methods). This is the phase picked up by a polariton hopping between cavities. Since polaritons must hop twice to interfere with the driving field in the first cavity, the stationary population depends on the round-trip phase 2(*φ*_1_–*φ*_2_). Figure 4a shows that 2(*φ*_1_–*φ*_2_)≈0 (modulo 2*π*) for the lowest and upper branches, irrespective of the driving strength. These branches correspond to the lowest and highest branches in Fig. 3a,b, where interference in the driven cavity is constructive. Note that for the lowest (resp. upper) branch, *φ*_1_–*φ*_2_≈−*π* (resp. 0), which is the characteristic phase relation of the antibonding (resp. bonding) mode. Interestingly, for the middle branch in Fig. 4a, *φ*_1_–*φ*_2_ varies from −*π* to 0. Therefore, the round-trip phase makes the interference in the driven cavity change from constructive to destructive and back to constructive for increasing intensity.

We performed power-dependent interferometry measurements to directly observe the predicted interaction-induced hopping phase. For this purpose, the cavity transmission was interfered with an expanded section of the excitation laser beam (see Methods). Next, we fitted cosine functions to the normalized interferogram in each cavity. Figure 4b shows the difference between the fitted phases, *φ*_1_–*φ*_2_, in good agreement with our calculations. Figure 4c–h shows representative density (left panels) and interferogram (right panels) plots along the middle branch (black squares in Fig. 4b). Figure 4c,d and g,h show a significant density in the driven cavity when *φ*_1_–*φ*_2_≈−*π* and *φ*_1_–*φ*_2_≈0, respectively; these are conditions of constructive interference. In contrast, Fig. 4e,f shows that the driven cavity is dark at the destructive interference condition *φ*_1_–*φ*_2_≈−π*/2*, that is, a round trip phase of −*π*. The observation of this density-dependent interference demonstrates that the hopping phase can be optically controlled through interactions.

## Discussion

Beyond the BHD, an interaction-controlled hopping phase in two-dimensional lattices could enable the exploration of BHMs with density-dependent gauge fields. The proposed extension relates to the seminal work by Aharanov and Bohm^24^ and Berry^25^, who realized that a nonzero phase acquired by a particle in a closed-loop trajectory implies the existence of a nonzero vector potential **A**. Specifically, the phase acquired when hopping from site *i* to *j* can be expressed as 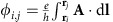, where *e* is the elementary charge^26^. Thus, synthethic magnetism^27^,^28^,^29^,^30^,^31^ and topologically non-trivial states^32^,^33^,^34^ could be achieved with photons in two-dimensional arrays of coupled nonlinear cavities by engineering an interaction-induced hopping phase.

## Methods

### Sample

The planar cavity was grown by molecular beam epitaxy and comprises a 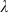 GaAs cavity between two Ga_0.9_Al_0.1_As/Ga_0.05_Al_0.95_As distributed Bragg reflectors with 26 and 30 pairs for the top and bottom one, respectively. One 80-Å-wide InGaAs quantum well with an exciton energy of 1480.7 meV is positioned at the centre of the cavity. Strong exciton-photon coupling leads to a Rabi splitting of 3.4 meV. The coupled microcavities are fabricated by electron beam lithography and dry etching of the planar cavity. Based on the linear transmission spectra, we estimate a polariton ground-state energy for each microcavity of 1476.6 meV and a linewidth of 37.5±1.5 μeV. Based on the polariton dispersion in the planar cavity (see Supplementary Figure 1 and Supplementary Note 1), we estimate a photon fraction |*C*|^2^=0.84±0.03 (*C* being the photonic Hopfield coefficient) at the driving energy of the experiments in Figs 3 and 4.

### Experiment

All experiments are performed at 4 K in transmission geometry, collecting the driving laser-transmitted intensity from the substrate side. The laser is a tunable MSquare Ti:Sapphire oscillator with <10 MHz linewidth. The excitation and collection objectives have a numerical aperture of 0.5 and 0.4, respectively. The excitation laser beam is linearly polarized parallel to the dimer axis (horizontal line at 0 μm in all density and interference colour plots).

We quantify the population in each cavity as follows. The transmitted intensity is recorded with a CCD camera without any spatial or spectral filtering. The counts detected within the left and right squares delimited by the grey solid lines in Fig. 1 are attributed to the driven and undriven cavities, respectively. The count rate for each cavity *n*_*j*_ is converted to the polariton population *N*_*j*_ via the following relation: 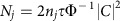. The factor of 2 takes into account that roughly half of the population decays in the direction opposite to the detector; *τ*=18 ps is the polariton lifetime, Φ is the detection efficiency (including collection) and |*C*|^2^ quantifies the fraction of polaritons that decay radiatively.

For the measurements in Fig. 4 we used a Mach-Zender interferometer as described next. The first beam splitter directed half of the power in the driving laser to the coupled cavities, and the other half of the power bypassed the cavities and served as a reference. The reference beam was expanded, making its beam waist at the position of the detector about three times the diameter of the cavity. The intensity in the reference beam was controlled with a neutral density filter. We recorded the transmitted intensity by the coupled cavities *I*_c_, and the intensity in the reference beam *I*_r_. Next, *I*_c_ and *I*_r_ were combined by a second beam splitter placed between the output of the cavities and the detector. We call the combined total intensity *I*_t_. The data were analysed with the two-beam interference equation 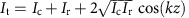. The quantity *kz* corresponds to the optical path difference between the two arms of the interferometer, which is controlled by the position of the second beam splitter and the alignment of the two beams. Figure 4d,f,h plots the cos(*kz*) term in colour as a function of space. We call this quantity, bounded between −1 and 1, the normalized interferogram. To retrieve the interaction-induced hopping phase in Fig. 4b, we repeated this procedure while scanning the driving power along all three branches. Next, we analysed the normalized interferogram as follows. We took cuts of the interferogram along vertical lines (dash-dotted lines in Fig. 4d,f,h) at a distance of ±0.5 μm from the centre of the dimer. The cuts at −0.5 μm correspond to the driven cavity, and the cuts at +0.5 μm correspond to the undriven cavity. To each cut we fitted a function of the form *A*_*j*_cos(*B*_*jy*_+*φ*_*j*_)+*C*_*j*_, where *A*_*j*_, *B*_*j*_, *φ*_*j*_ and *C*_*j*_ are fit parameters corresponding to the *j*th cavity (*j*=1, 2), and *y* is the vertical dimension. Figure 4b plots the difference between the fitted phases *φ*_1_–*φ*_2_. The behaviour reported for *φ*_1_–*φ*_2_ at ±0.5 μm is robust over distances greater than 1 μm with respect to the centre of the dimer. For larger distances, the phase patterns in Fig. 4f,h exhibit dislocations where the mode intensity vanishes near the walls of the driven cavity. The origin of these dislocations is the presence of parasitic scattered laser light, which interferes with the weak cavity transmission at the detector. Due to the highly nonlinear transmission through the driven cavity, the contribution of the parasitic light can be more than 2 orders of magnitude greater at high irradiance than at low irradiance. The contribution of the parasitic light to the measured phase patterns is only significant in the regions of vanishing mode intensity at the edges of the driven cavity. Hence, the measured phase patterns in these regions do not reflect the intracavity field phase only. For these reasons we consistently analyse the phase patterns in Fig. 4 far from these artefacts and near the centre of the dimer, that is, at ±0.5 μm.

### Calculations

For all calculations we seek the stationary solutions 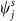 (*j*=1, 2) to the differential equation (1). We start by inserting the ansatz 
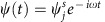
 in equation (1). This leads to the algebraic equations


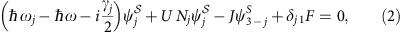


where 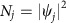 are the mode populations. The populations are obtained by writing the above equations as a polynomial in powers of *N*_2_, calculating the roots of that polynomial, and then inserting the solutions in the remaining equation to obtain *N*_1_. The phase difference between the intracavity fields, *φ*_1_–*φ*_2_, is calculated by inserting the populations in the supplementary equations 
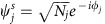
. Finally, we assess the stability of the stationary solutions by analysing the spectrum of small fluctuations in their vicinity, that is, 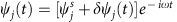. This is performed following the procedure outlined by Sarchi *et al.*^16^. Sections 2 and 3 from ref. 16 describe the physics of the model we employ throughout this manuscript (equation (1)), including the various kinds of stable solutions and instabilities that the two coupled nonlinear modes support. However, the analysis therein is restricted to the populations of the two modes and not to their relative phases. The phase analysis in Fig. 4a and the counting of the number of modes in Fig. 2 are results from the present work.

Based on Lorentzian fits to the measured linear spectrum, we set 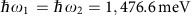, *γ*_1_=*γ*_2_=*γ*=37.5 μeV, and *J*=179 μeV for all calculations. *U*=0.07 μeV was set to match the multistability experiments in Fig. 3. Taking the cross-sectional area *A* of each cavity into account, the two-dimensional polariton-polariton interaction constant is 0.8 μeV *μ*m^2^. Dividing by |*X*|^2^=0.16^2^ we get 30 μeV *μ*m^2^ for the pure exciton-exciton interaction constant. A similar value for the exciton-exciton interaction constant has been theoretically estimated in ref. 21.

### Data availability

The data that support the findings of this study are available from the corresponding author upon request.

## Additional information

**How to cite this article:** Rodriguez, S. R. K. *et al.* Interaction-induced hopping phase in driven-dissipative coupled photonic microcavities. *Nat. Commun.* 7:11887 doi: 10.1038/ncomms11887 (2016).

## Supplementary Material

Supplementary Information Supplementary Figures 1-4 and Supplementary Notes 1-4.
