# Supplementary material for: Interaction-induced hopping phase in driven-dissipative coupled photonic microcavities
Source: Nat Commun. 2016 Jun 16;7:11887. doi: 10.1038/ncomms11887 (PMC4912632; doi:10.1038/ncomms11887)
Supplement: Supplementary Information — Supplementary Figures 1-4 and Supplementary Notes 1-4. [file ncomms11887-s1.pdf]

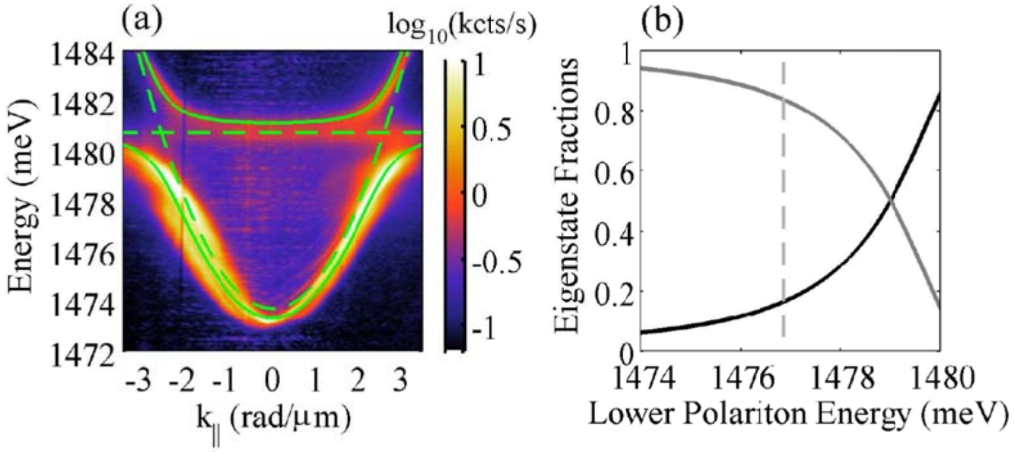

Supplementary Figure 1. **Polariton dispersion and eigenstate fractions.** (a) Non-resonant photoluminescence measurements of an effectively two-dimensional cavity. The horizontal and parabolic dashed lines indicate the bare exciton and photon energies, respectively. The solid lines are the upper and lower polaritons formed by strong exciton-photon coupling. (b) For the lower polariton, the exciton fraction is shown as a black line and the photon fraction is shown as a gray line, both as a function of the lower polariton energy.

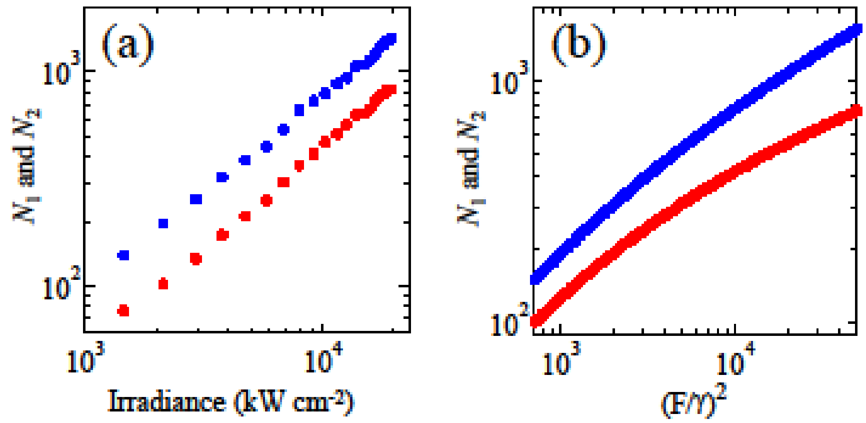

Supplementary Figure 2. **Monostable regime.** (a) Measurements, and (b) calculations of the population N_j (with j=1,2) in each cavity at a driving energy of 1476.36 meV. The blue color corresponds to the driven cavity, and the red color corresponds to the undriven cavity. For the calculations, F is the driving amplitude and γ is the bare cavity linewidth.

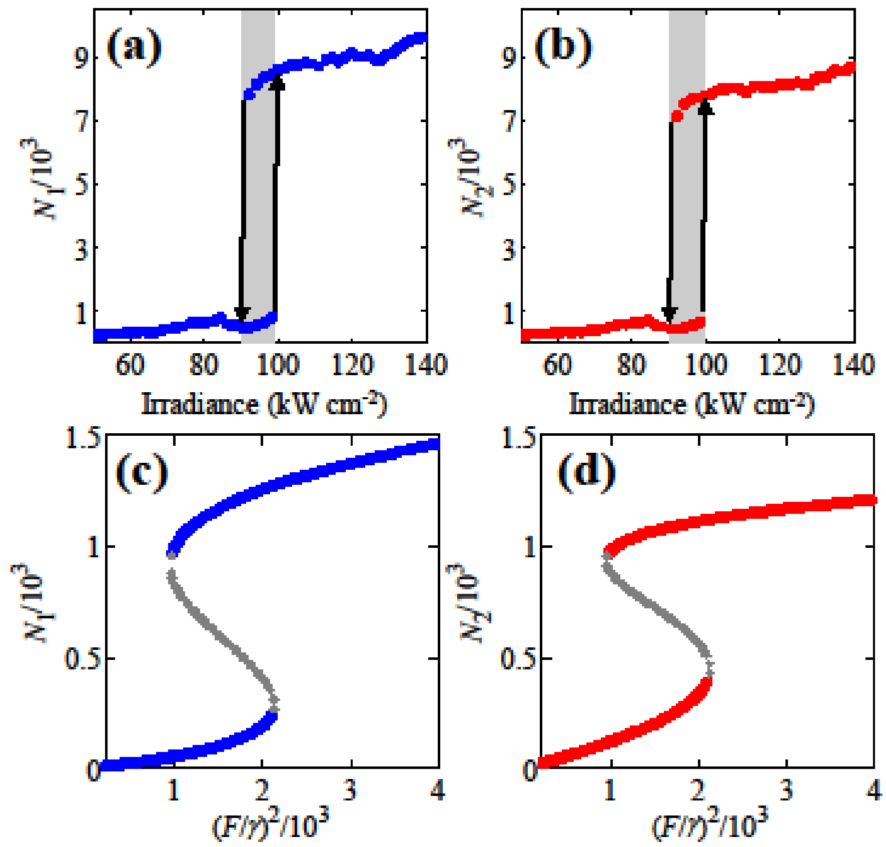

Supplementary Figure 3. **Bistability.** (a) and (b) show the measured populations in the driven and undriven cavities respectively, when driving the photonic molecule at 1476.46 meV; (c) and (d) show the corresponding calculated populations using the model described in the text. Blue and red data points are stable solutions while gray ones are unstable solutions.

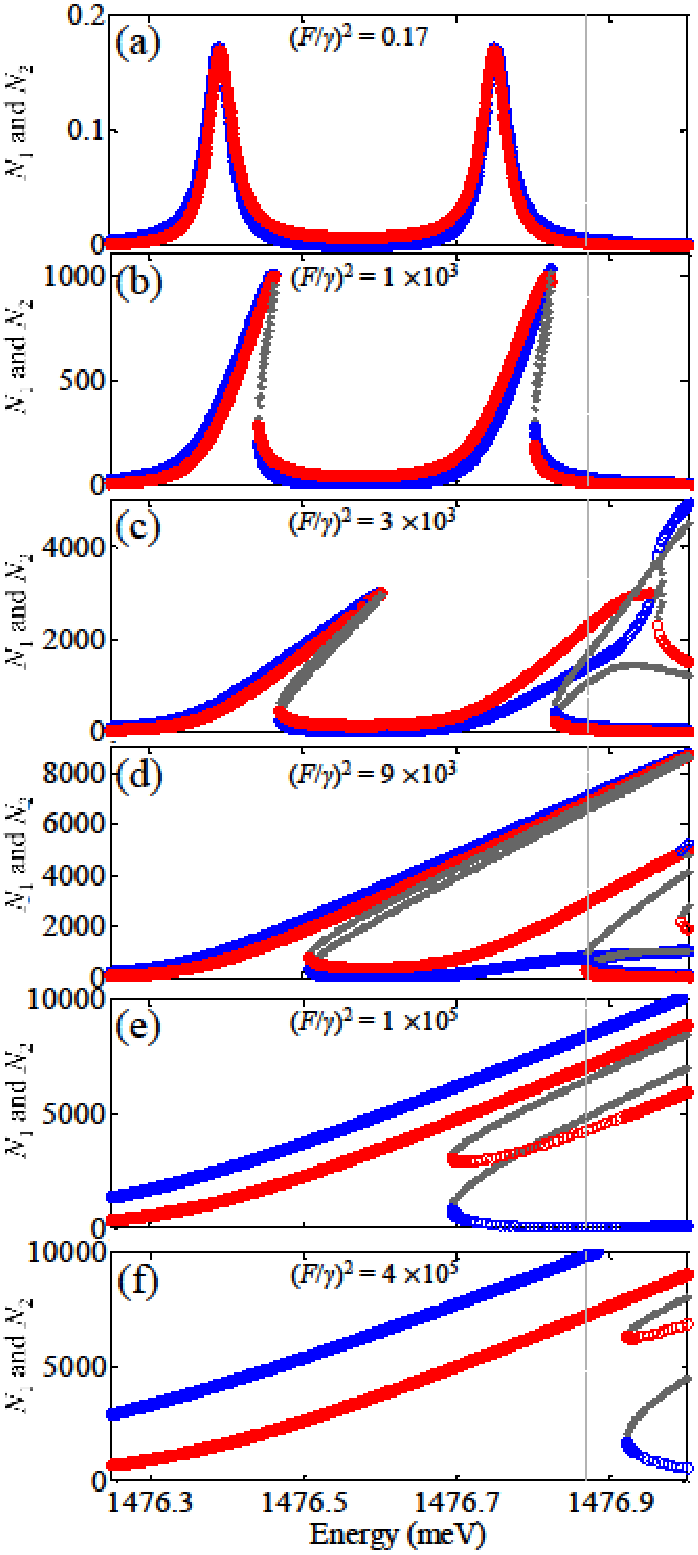

Supplementary Figure 4. **Cavity-resolved power-dependent spectrum.** Calculated population of the driven cavity N_1 (blue lines) and of the undriven cavity N_2 (red lines) for a normalized driving power $(F/\gamma)^2$ indicated at the top of each panel. F is the driving amplitude and γ is the bare cavity linewidth. Open circles indicate parametrically unstable modes, and gray lines indicate unstable solutions. The vertical line in all panels indicates the driving energy used for the experiments in Fig. 3 and Fig. 4 of the main manuscript.

Supplementary Note 1. Exciton polaritons in the 2D cavity

The microstructure investigated in the main text is fabricated by etching a two-dimensional (2D) cavity into the shape of two coupled cavities [Fig. 1(a) in the main text]. Some parts of the 2D cavity are etched on a much larger scale (e.g. 200 microns), leaving behind structures which display the same optical properties as the unetched cavity. Here we analyze the exciton polariton dispersion in such an effectively 2D cavity, where the lateral confinement energy is negligible. Through this analysis, we estimate the exciton and photon fractions of polaritons in the microstructure.

Supplementary Figure 1 shows non-resonant photoluminescence measurements of the aforementioned effectively 2D cavity. The cavity is pumped by a continuous wave laser with energy of 1.61 eV. The emitted intensity is analyzed spectrally and angularly by imaging the back focal plane of the objective onto a CCD camera. Supplementary Figure 1(a) shows the emitted intensity (in log scale) in color as a function of the emission energy and wave vector component parallel to the quantum well plane. The measurements show the avoided resonance crossing characteristic of exciton polaritons, formed by the strong coupling between cavity photons and quantum well excitons.

We model the polariton system with the following effective 2×2 Hamiltonian,

$$H = \begin{pmatrix} E_X & \Omega \\ \Omega & E_C(k_{\|}) \end{pmatrix} \quad (1)$$

E_X and E_C ($k_{\|}$) are the bare exciton and photon energy, respectively; these are the horizontal and parabolic dashed lines Supplementary Figure 1(a). The off-diagonal terms of the Hamiltonian quantify the strength of the exciton-photon coupling – a fit parameter here set to Ω = 1.7 meV. We calculate the polariton dispersion, i.e. the energy of the mixed states as a function of $k_{\|}$ by diagonalizing the Hamiltonian. The solid lines in Supplementary Figure 1(a) indicate the calculated upper and lower polariton dispersion. The polariton eigenstates can be expressed as $|P\rangle = X(k_{\|})|X\rangle + C(k_{\|})|C\rangle$ with $|X\rangle$ and $|C\rangle$ the exciton and cavity photon states. $X(k_{\|})$ and $C(k_{\|})$ are the Hopfield coefficients of the eigenvector associated with the eigenvalues of the Hamiltonian (the polariton energy). The magnitude squared of these coefficients, i.e. $|X(k_{\|})|^2$ and $|C(k_{\|})|^2$, are the eigenstate fractions characterizing the polariton admixture. In Supplementary Figure 1(b) we plot the exciton fraction as a black line and the photon fraction as a gray line, both for the lower polariton as a function of its energy. The vertical dashed line in Supplementary Figure 1(b) indicates the driving energy of the experiments in Figure 3 and Figure 4 of the main text. At this energy, the lower polariton has a photon fraction of $|C(k_{\|})|^2 = 0.84$.

Supplementary Note 2. Monostable regime

For a driving energy below the bonding mode energy, each cavity comprising the photonic molecule (PM) exhibits a single input-output branch which is stable for all driving strengths.

In Supplementary Figure 2 we show experiments and calculations when driving the left cavity at an energy of 1476.36, red-detuned from the peak bonding energy. Both measurements and calculations show that there is a single input-output branch for all powers. In addition, for very strong driving the calculation shows that the population in the undriven cavity saturates. This power limiting effect is due to the fact that all modes are at energies above the driving energy, and that they nonlinearly blue-shift for increasing driving strength due to repulsive interactions. As discussed in Ref. 16 of the main manuscript, this configuration is often called the optical limiter.

Supplementary Note 3. Bistable regime

For a driving energy between the bonding and antibonding mode energies, the PM exhibits up to two stable branches at the same irradiance, i.e. bistability. Supplementary Figure 3 shows an example of bistability in the intensity-dependent population of each cavity when driving the left cavity with an energy of 1476.46 meV. The shaded areas indicate the bistable region. The calculations obtained using the model equations 1 in the main text, and shown in Supplementary Figures 3(c,d) give good overall description of this bistable regime.

Supplementary Note 4. Calculated Spectrum

Supplementary Figure 4 shows the calculated spectrum of the PM for various driving powers. Blue lines indicate the population in the driven cavity, red lines indicate the population in the undriven cavity, open circles indicate parametrically unstable modes, and gray lines indicate unstable modes. Supplementary Figure 4(a) shows the spectrum of the PM in the linear regime (weak driving), here obtained for $(F/\gamma)^2 = 0.17$. The spectrum in Supplementary Figure 4 (a) is in good agreement with the experimental result in Figure 1(a) of the main manuscript. This agreement validates the model parameters retrieved from the Lorentzian fits to the bonding and antibonding resonances in Fig. 1(a). In Supplementary Figure 4 (b) we calculate the spectrum for $(F/\gamma)^2 = 10^3$, where single mode bistability is observed. This corresponds to the regime studied in Supplementary Section 3. In Supplementary Figure 4(c) obtained for $(F/\gamma)^2 = 3\times10^3$, tristability appears at high energies. For the driving energy of the experiments in Figure 3 and Figure 4 of the main manuscript [indicated by the dashed line in all panels of Supplementary Figure 4] there are two stable modes. In Supplementary Figure 4(d) obtained for $(F/\gamma)^2= 9\times10^3$, the PM is tristable at the driving energy of the experiments in Fig. 3 and Fig. 4. In Supplementary Figure 4 (e) obtained for $(F/\gamma)^2= 10^5$, the PM becomes bistable again at the same energy. The driving power in Supplementary Figure 4(e) is close to the dip in population of the driven cavity due to destructive interference. Notice how the lower branch of the driven cavity lies very close to zero population for a wide range of driving energies. This result suggests that the interaction-induced destructive interference effect described in the main manuscript is robust to relatively large changes in energy (up to several linewidths). Finally, Supplementary Figure 4 (f) obtained for $(F/\gamma)^2= 4\times10^5$ shows that for very strong driving the PM becomes monostable at the energy of the dashed line. Thus, the series of calculations in Supplementary Figure 4 illustrates how the spectrum of the PM evolves as the driving power and interaction energy increase, and how the number of stable modes changes.
